# Changes in patient-reported outcomes and objective assessments based on baseline symptom severity in SURMOUNT-OSA: post-hoc analyses

**DOI:** 10.1093/sleep/zsag184

**Published:** 2026-07-07

**Authors:** Chisom Kanu, Shraddha Shinde, Beverly Falcon, Josef Bednarik, Cathy Chang Xie, Kevin Trice, Terri Weaver

**Affiliations:** Eli Lilly and Company, Indianapolis, IN, United States; Eli Lilly and Company, Indianapolis, IN, United States; Eli Lilly and Company, Indianapolis, IN, United States; Eli Lilly and Company, Indianapolis, IN, United States; Eli Lilly and Company, Indianapolis, IN, United States; Eli Lilly and Company, Indianapolis, IN, United States; School of Nursing, University of Pennsylvania, Philadelphia, PA, United States; College of Nursing, University of Illinois Chicago, Chicago, IL, United States

**Keywords:** obstructive sleep apnea, tirzepatide, patient-reported outcome measures, OSA symptoms

## Abstract

**Study Objectives:**

These post-hoc analyses of SURMOUNT-OSA evaluated patient-reported outcome measures (PROMs) and objective assessments, considering participants’ baseline characterization of fatigue, sleepiness, snoring, and sleep quality.

**Materials and Methods:**

SURMOUNT-OSA included two 52-week randomized, placebo-controlled studies of tirzepatide (10/15 mg) in participants with moderate-to-severe obstructive sleep apnea (OSA) and obesity (Study 1: participants not using positive airway pressure [PAP]; Study 2: participants using PAP). Participants were grouped by self-reported baseline severity of fatigue (Non-fatigued/Fatigued), sleepiness (Non-sleepy/Sleepy), snoring (Non-snoring/Snoring), and sleep quality (Good/Poor). Changes in PROMs and objective assessments from baseline to Week 52 were assessed per subgroup.

**Results:**

In both studies, tirzepatide treatment was consistently associated with greater improvements in PROMs and objective assessments than placebo. In Study 1, tirzepatide-treated participants in the Fatigued subgroup had numerically greater improvements versus Non-fatigued subgroup in Functional Outcomes of Sleep Questionnaire Activity Level (least-square means [LSM] difference from placebo: 0.33 vs. 0.05), SF-36v2 Vitality, (8.5 vs. 1.0), and SF-36v2 General Health scores (6.2 vs. 1.4). The Sleepy subgroup showed numerically greater improvements versus Non-sleepy subgroup in Patient-Reported Outcomes Measurement Information System (PROMIS) Short-Form Sleep-Related Impairment 8a scores (LSM difference: –7.1 vs. –1.6) while the Poor sleep quality subgroup had numerically greater improvements versus Good sleep quality subgroup in PROMIS Short-Form Sleep Disturbance scores (LSM difference: –5.8 vs. –1.2). Changes in PROMs were similar in both snoring subgroups. Study 2 patterns were generally consistent with Study 1. Tirzepatide treatment was associated with similar improvements in objective assessments (apnea-hypopnea index, sleep apnea-specific hypoxic burden, weight, OSA remission) across all symptom severity subgroups in both studies.

**Conclusions:**

Across subgroups, tirzepatide treatment was associated with greater improvements in PROMs than placebo. Participants with worse fatigue, sleepiness, and sleep quality at baseline generally had larger improvements on related PROMs than those with milder symptoms. Tirzepatide treatment was also associated with greater improvements in objective assessments versus placebo, though these changes were generally similar across baseline symptom severity subgroups.

**Clinical Trial Registration:**

Registered at ClinicalTrials.gov under “Obstructive Sleep Apnea Master Protocol GPIF: A Study of Tirzepatide (LY3298176) in Participants With Obstructive Sleep Apnea (SURMOUNT-OSA)”; https://clinicaltrials.gov/study/NCT05412004; registration number: NCT05412004

## Graphical Abstract

**Graphical abstract:**
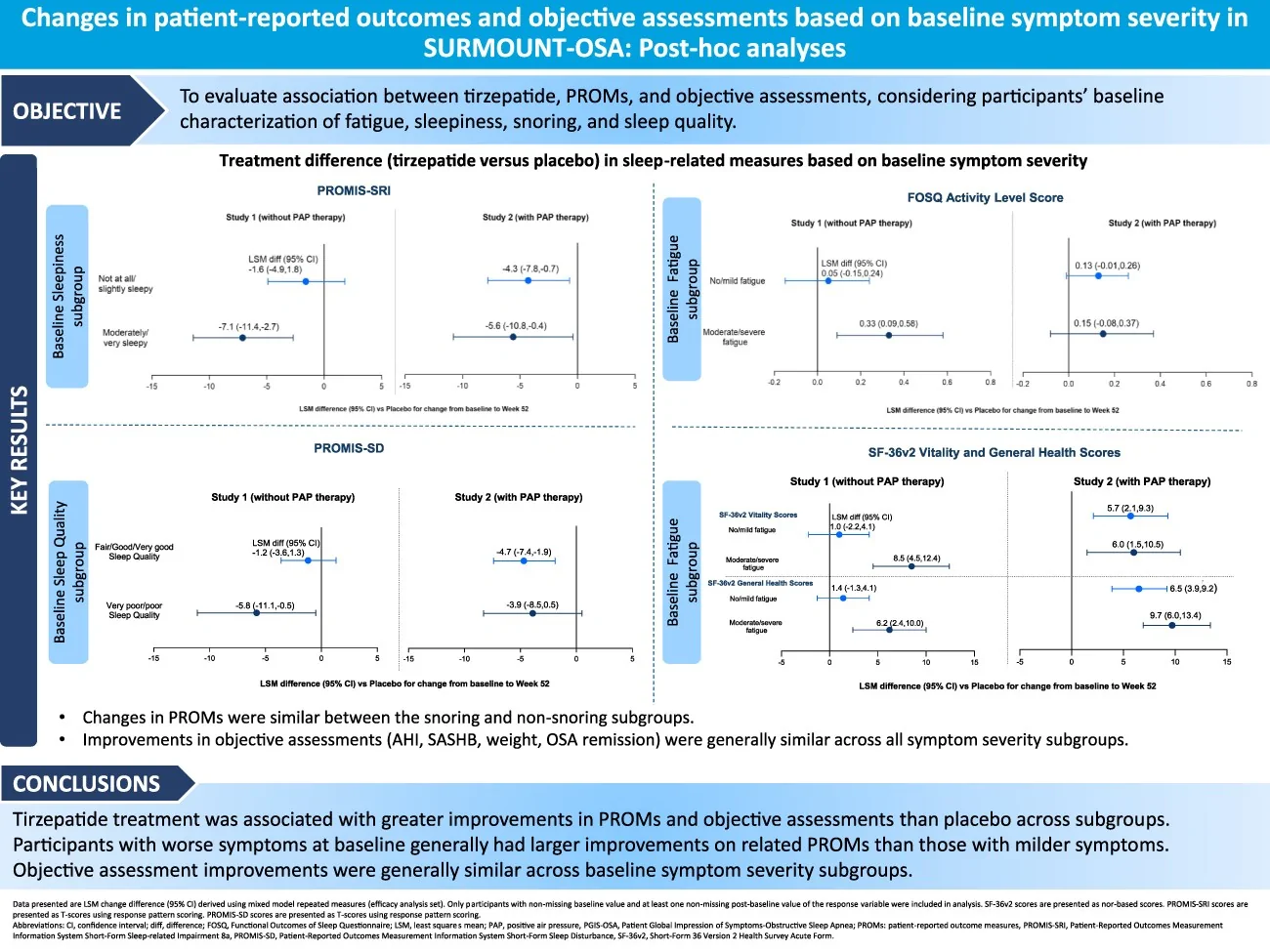

**Statement of Significance**

Obstructive sleep apnea (OSA) is a complex, heterogenous condition, with some patients experiencing greater symptom burden than others. These post-hoc analyses of SURMOUNT-OSA evaluated the association between treatment outcomes and participants’ baseline severity of fatigue, sleepiness, snoring, and sleep quality. Tirzepatide treatment was generally associated with greater improvements in patient-reported outcomes measures and objective assessments than placebo. While improvements in objective assessments across symptom severity subgroups were largely similar, participants with severe baseline symptoms generally reported greater improvements in sleep-related impairment, perceived sleep disturbance, activity, vitality, and general health, than those with milder baseline symptoms. These results provide relevant context for physicians treating patients with moderate-to-severe OSA and obesity who exhibit varying symptom profiles and sleep-related concerns.

## Introduction

Globally, nearly 1 billion adults aged 30 to 69 years have mild-to-severe obstructive sleep apnea (OSA; apnea-hypopnea index [AHI] ≥5 events per hour), and 425 million adults aged 30 to 69 years have moderate-to-severe OSA (AHI ≥15 events per hour) globally [1]. Its occurrence is higher among individuals with obesity (40 per cent to 70 per cent) compared with the general population (17 per cent) [2]. OSA is associated with frequent awakenings and fragmented sleep cycles, which can hamper restful sleep, leading to poor sleep quality and tiredness during the day [3, 4]. It is a major cause of excessive daytime sleepiness, which contributes to reduced quality of life, impaired work performance, and increased risk of motor vehicle accidents [3, 4]. While the commonly reported symptoms of OSA include snoring (up to 95% of patients), excessive daytime sleepiness (up to 58%), fatigue (40%–42%), and poor sleep quality (up to 19.5%) [5–9], the extent and combination of these symptoms differ from person to person regardless of their AHI severity, with some patients experiencing greater symptom burden than others [9].

Tirzepatide is a once-weekly glucose-dependent insulinotropic polypeptide and glucagon-like peptide-1 receptor agonist. It has been approved in several countries for the treatment of type 2 diabetes and obesity [10, 11]. Tirzepatide is the first medication approved by the US Food and Drug Administration for the treatment of moderate-to-severe OSA in adults with obesity [12]. In SURMOUNT-OSA (NCT05412004) in participants with moderate-to-severe OSA and obesity treatment with tirzepatide led to clinically meaningful changes in sleep-disordered breathing and alleviation of perceived sleep disturbance and sleep-related impairment, as well as reductions in common OSA-related cardiovascular risk factors [13]. Additionally, participants reported significant improvements in functional status, HRQoL, and symptom severity following tirzepatide treatment [14].

Although alternative treatment options exist, there may be some limitations on their use in people with moderate or severe OSA and obesity. For instance, most of these treatments do not consider individual heterogeneity, diverse risk factors, pathophysiological causes, symptoms, and outcomes associated with the condition [15, 16]. Understanding the effect of treatments in patients presenting with different symptom severity at the onset of treatment can guide individualized treatment strategies and may improve outcomes. These post-hoc analyses of SURMOUNT-OSA evaluated the association between tirzepatide, patient-reported outcome measures (PROMs), and objective assessments based on participants’ baseline characterization of their sleepiness, fatigue, snoring, and sleep quality.

## Materials and methods

### Study design and population

SURMOUNT-OSA included two 52-week, multicenter, randomized, placebo-controlled phase 3 trials (Study 1 and Study 2) that evaluated the efficacy and safety of the maximum tolerated dose (MTD; 10 mg or 15 mg) of once-weekly tirzepatide among adults with moderate-to-severe OSA (AHI ≥15 events per hour, scored using AHI-4 per cent [≥4% oxygen desaturation]) and obesity (body mass index [BMI] ≥30 kg/m^2^) [13]. Study 1 had participants who were unable or unwilling to use positive airway pressure (PAP) therapy, while Study 2 had those who had been using PAP therapy for at least three consecutive months at screening and who planned to continue during the trial. Participants were randomly assigned (1:1) to receive tirzepatide MTD or placebo for 52 weeks. All participants received lifestyle counseling (including nutritional counseling, calorie-deficit diet, and physical activity) [13]. Participants in Study 2 were required to suspend PAP therapy for 7 days before polysomnography and PROM assessments at baseline and Week 52 to minimize the confounding effect of PAP therapy on assessments. The study design, procedures, and eligibility criteria have been previously published [13].

The trials were conducted in accordance with Good Clinical Practice guidelines and the principles of the Declaration of Helsinki. Each participating site received approvals from an independent ethics committee or institutional review board. All participants provided written informed consent before trial participation.

### Study measures and assessments

At baseline and Week 52, participants self-rated their OSA symptom severity (fatigue, sleepiness, snoring) and sleep quality over the past 7 days using the Patient Global Impression of Status (PGIS)-OSA scales and Item 8 of the Patient-Reported Outcomes Measurement Information System (PROMIS) Short-Form v1.0 Sleep Disturbance 8b (PROMIS-SD), which assesses sleep quality. Participants were categorized into 2 subgroups based on their baseline responses to each global impression item:

1. PGIS-OSA fatigue: Non-Fatigued (“no/mild” fatigue) and Fatigued (“moderate/severe” fatigue);
2. PGIS-OSA Sleepiness: Non-sleepy (“not at all/slightly sleepy”) and Sleepy (“moderately/very sleepy”);
3. PGIS-OSA Snoring: Non-snoring (sleep “not at all/slightly affected” by snoring) and Snoring (sleep “moderately/very affected” by snoring); and
4. Item 8 of the PROMIS-SD: Good sleep quality (“fair/good/very good” sleep quality) and Poor sleep quality (“very poor/poor” sleep quality).

Changes from baseline to Week 52 in each of these symptom subgroups were assessed for PROM scores conceptually related to these baseline symptoms as well as key objective assessments (additional details in Table 1 and Supplementary Table S1):

**Table 1:** Summary of study measures and assessments

Symptom scales	Baseline groups based on symptom scales	PROMs assessed for changes from baseline to Week 52 and score interpretation
PGIS-OSA Fatigue	Non-fatigued (no/mild fatigue) Fatigued (moderate/severe fatigue)	FOSQ-10 and FOSQ Activity level: Higher scores indicate improvement SF-36v2 (General health and vitality domains): Higher scores indicate better levels of function and/or better health PGIC-OSA fatigue: Responses of A little better/Much better indicate improvement
PGIS-OSA Sleepiness	Non-sleepy (not at all/slightly sleepy) Sleepy (moderately/very sleepy)	ESS: Higher scores indicate greater daytime sleepiness PROMIS-SRI: Higher scores indicate more sleep-related impairment FOSQ vigilance: Higher scores indicate improvement PGIC-OSA sleepiness: Responses of A little less/Much less sleepiness indicate improvement
PGIS-OSA Snoring	Non-snoring (Sleep not at all/slightly affected by snoring) Snoring (Sleep moderately/very affected by snoring)	PGIC-OSA snoring: Responses of A little less/Much less affected indicate improvement
Sleep quality (Item 8a of PROMIS-SD)	Good sleep quality (fair/good/very good) Poor sleep quality (very poor/poor)	PROMIS-SD: Higher scores indicate more sleep disturbance PGIC-OSA sleep quality: Responses of A little better/Much better sleep quality indicate improvement

Abbreviations: ESS, Epworth Sleepiness Scale; FOSQ, Functional Outcomes of Sleep Questionnaire; OSA, obstructive sleep apnea; PGIC, Patient Global Impression of Change; PGIS, Patient Global Impression of Status; PROM, patient-reported outcome measure; PROMIS-SRI, PROMIS Short-Form Sleep-related Impairment; SF-36v2, Short-Form 36 Version 2 Health Survey, Acute Form.

(1) PROMIS Short-Form Sleep-related Impairment 8a (PROMIS-SRI), which assesses alertness, sleepiness, and tiredness during usual waking hours. Higher scores indicate more sleep-related impairment. Scores were examined by PGIS Sleepiness subgroups.

(2) PROMIS-SD, which assesses sleep quality, sleep depth, and restoration associated with sleep. Higher scores indicate more sleep-related disturbance. Scores were examined by PGIS Sleep Quality subgroups.

(3) Epworth Sleepiness Scale (ESS) assesses participants’ usual chances of dozing in different daytime situations. Higher scores indicate greater daytime sleepiness. Scores were examined by PGIS Sleepiness subgroups.

(4) Functional Outcomes of Sleep Questionnaire (FOSQ) evaluates the effect of disorders associated with excessive daytime sleepiness on daily functioning in adults in various domains. The 10-item subset (FOSQ-10) assesses the impact of sleep on daily activities. Higher scores indicate better functional status. FOSQ-10 and FOSQ Activity Level domain scores were examined by PGIS Fatigue subgroups. FOSQ Vigilance domain score was examined by PGIS Sleepiness subgroups.

(5) Short-Form 36 Health Survey, Version 2 (SF-36v2) acute form is a generic measure that assesses of quality of life across several domains. Higher scores indicate better levels of function or better health. The General Health and Vitality domain scores were examined by PGIS Fatigue subgroups.

(6) Patient Global Impression of Change (PGIC)-OSA scales: PGIC-OSA Fatigue, PGIC-OSA Sleepiness, PGIC-OSA Sleep Quality, and PGIC-OSA Snoring were examined by subgroups of the corresponding PGIS symptom.

(7) Objective assessments included percent change from baseline in AHI, body weight, and sleep apnea-specific hypoxic burden (SASHB) as well as percentage of participants achieving OSA remission (AHI <5 events/hour).

### Statistical analyses

These post-hoc analyses were conducted in the efficacy analysis set with all randomized participants who were exposed to at least 1 dose of study treatment, and with non-missing baseline PROM values for grouping. Data after discontinuation of study treatments were excluded. For Study 1, data after initiating PAP therapy were also excluded. The changes from baseline to Week 52 in PROMs were computed using mixed model repeated measures (MMRM) [17], controlling for baseline value of the PROM analyzed, geographic region (US/outside of US), baseline OSA severity (not severe: AHI <30 events/hour; severe [AHI ≥30 events/hour]), sex, treatment, time, and the interaction between treatment and time as covariates. Percent change from baseline to Week 52 in AHI and bodyweight were computed using MMRM with similar covariates. Percent change from baseline to Week 52 in SASHB was computed using MMRM with log transformation. The PGIC-OSA overall change was summarized at Week 52 for tirzepatide MTD and placebo arms by the PGIS-OSA subgroups at baseline. Participants with non-missing PGIC data at week 52 were included for PGIC analyses. The percentage of participants with OSA remission at Week 52 was estimated after imputing missing AHI data at Week 52 using the multiple imputation method. The odds ratio for remission was derived from logistic regression with the imputed datasets after controlling for baseline AHI, geographic region, sex, and treatment. The results are pooled using Rubin’s rules [18].

Summary statistics included mean and standard deviation (SD) for continuous measures, and frequency, and percentages for categorical measures. Least-square means (LSM), model-based estimate (MBE), and standard errors (SE) for the change and percent change from baseline measurements were derived from the MMRM. Treatment comparisons were presented as the LSM or MBE change and percent change difference with 95% confidence intervals (CI). For OSA remission, odds ratios with 95% CI were reported. Given the post-hoc, exploratory nature of the study and the limited size of the subgroups, no formal hypothesis testing (including statistical tests for treatment-by-subgroup interactions) was conducted. Accordingly, results are interpreted descriptively, and any subgroup comparisons are intended to be hypothesis-generating rather than definitive.

## Results

### Baseline characteristics

Baseline demographics and clinical characteristics of participants were generally balanced between subgroups (Study 1: mean age range: 46.8 to 48.3 years; BMI range: 38.5 to 39.7 kg/m^2^; Study 2: mean age range: 49.7 to 52.2 years; BMI range: 37.1 to 40.0 kg/m^2^; Tables 2 and 3). In general, the baseline AHI, and PROMIS-SD, PROMIS-SRI, and ESS total scores were numerically higher among participants with severe symptoms at baseline in both studies.

**Table 2:** Participants’ demographics and baseline characteristics (Study 1—participants not using PAP therapy)

	PGIS-OSA fatigue		PGIS-OSA sleepiness		PGIS-OSA snoring		Sleep quality	
Characteristic	Non-fatigued *N* = 113	Fatigued *N* = 81	Non-sleepy *N* = 119	Sleepy *N* = 75	Non-snoring *N* = 103	Snoring *N* = 91	Good sleep quality *N* = 148	Poor sleep quality *N* = 50
Male, *n* (%)	86 (76.1)	46 (56.8)	87 (73.1)	45 (60.0)	67 (65.0)	65 (71.4)	98 (66.2)	37 (74.0)
Age, years	48.3 (11.4)	46.8 (11.3)	47.9 (11.6)	47.3 (11.1)	48.2 (12.1)	47.1 (10.5)	47.6 (11.1)	47.9 (12.1)
Age categories (years), *n* (%)								
<50	58 (51.3)	47 (58.0)	64 (53.8)	41 (54.7)	50 (48.5)	55 (60.4)	78 (52.7)	29 (58.0)
≥50	55 (48.7)	34 (42.0)	55 (46.2)	34 (45.3)	53 (51.5)	36 (39.6)	70 (47.3)	21 (42.0)
Race, *n* (%)								
American Indian or Alaska native	13 (11.5)	2 (2.5)	12 (10.1)	3 (4.0)	10 (9.7)	5 (5.5)	12 (8.1)	3 (6.0)
Asian	30 (26.5)	16 (19.8)	36 (30.3)	10 (13.3)	21 (20.4)	25 (27.5)	37 (25.0)	10 (20.0)
Black or African American	5 (4.4)	3 (3.7)	6 (5.0)	2 (2.7)	4 (3.9)	4 (4.4)	6 (4.1)	2 (4.0)
White	63 (55.8)	60 (74.1)	64 (53.8)	59 (78.7)	67 (65.0)	56 (61.5)	91 (61.5)	35 (70.0)
Multiple	2 (1.8)		1 (0.8)	1 (1.3)	1 (1.0)	1 (1.1)	2 (1.4)	
BMI, kg/m^2^	38.8 (7.0)	39.2 (6.9)	38.5 (6.9)	39.7 (7.0)	39.2 (7.6)	38.7 (6.2)	39.0 (7.0)	38.8 (6.7)
BMI categories (kg/m^2^), *n* (%)								
<35	37 (32.7)	28 (34.6)	44 (37.0)	21 (28.0)	36 (35.0)	29 (31.9)	49 (33.1)	17 (34.0)
≥35 and < 40	38 (33.6)	24 (29.6)	38 (31.9)	24 (32.0)	29 (28.2)	33 (36.3)	48 (32.4)	15 (30.0)
≥40	38 (33.6)	29 (35.8)	37 (31.1)	30 (40.0)	38 (36.9)	29 (31.9)	51 (34.5)	18 (36.0)
Weight (kg)	114.3 (24.7)	113.4 (22.4)	112.6 (23.6)	116.1 (23.9)	113.6 (24.7)	114.3 (22.8)	114.0 (23.4)	113.9 (24.3)
OSA severity, *n* (%)								
No Apnea		1 (1.3)	1 (0.8)		1 (1.0)		1 (0.7)	
Mild	3 (2.7)		3 (2.5)		3 (2.9)		3 (2.0)	
Moderate	30 (26.5)	27 (33.8)	33 (27.7)	24 (32.4)	31 (30.4)	26 (28.6)	44 (29.7)	17 (34.7)
Severe	80 (70.8)	52 (65.0)	82 (68.9)	50 (67.6)	67 (65.7)	65 (71.4)	100 (67.6)	32 (65.3)
Missing		1		1	1			1
AHI (events/hour)	52.6 (29.2)	54.4 (32.8)	51.8 (29.5)	55.8 (32.6)	48.4 (29.8)	58.8 (30.9)	51.6 (29.9)	56.1 (33.0)
ESS total score	9.2 (5.0)	12.6 (5.0)	8.7 (4.4)	13.9 (5.0)	8.8 (4.8)	12.7 (5.1)	10.1 (5.2)	12.2 (5.2)
FOSQ-10 Total score	16.7 (2.6)	13.6 (2.5)						
FOSQ Vigilance score			3.4 (0.6)	2.8 (0.7)				
FOSQ Activity Level	3.4 (0.5)	2.7 (0.6)						
SF-36v2 General health	48.7 (8.6)	43.9 (8.2)						
SF-36v2 Vitality	51.9 (8.0)	41.3 (7.4)						
PROMIS-SRI^a^	50.2 (7.4)	59.8 (6.0)	50.4 (7.6)	60.2 (5.3)	51.2 (8.4)	57.5 (6.9)	52.2 (8.1)	59.6 (6.4)
PROMIS-SD^a^	51.6 (6.6)	57.0 (6.0)	52.1 (7.0)	56.7 (5.8)	51.5 (7.1)	56.6 (5.7)	51.4 (5.9)	61.0 (4.1)

Data are presented as mean (SD), unless specified otherwise. Grey cells indicate missing values or values not reported.

^a^T-Score using response pattern scoring

Abbreviations: AHI, apnea-hypopnea index; BMI, body mass index; ESS, Epworth Sleepiness Scale; FOSQ, Functional Outcomes of Sleep Questionnaire; MTD, maximum tolerated dose; n, number of participants; N, total number of participants in the subgroup; PAP, positive air pressure; PGIS, Patient Global Impression of Status; PROMIS-SRI, Patient-Reported Outcomes Measurement Information System Short-Form Sleep-related Impairment 8a; PROMIS-SD, Patient-Reported Outcomes Measurement Information System Short-Form Sleep Disturbance.

**Table 3:** Participants’ demographics and baseline characteristics (Study 2—with PAP therapy)

	PGIS-OSA fatigue		PGIS-OSA sleepiness		PGIS-OSA snoring		Sleep quality	
Characteristic	Non-fatigued *N* = 105	Fatigued *N* = 88	Non-sleepy *N* = 110	Sleepy *N* = 83	Non-snoring *N* = 87	Snoring *N* = 106	Good sleep quality *N* = 115	Poor sleep quality *N* = 88
Male, *n* (%)	81 (77.1)	61 (69.3)	86 (78.2)	56 (67.5)	62 (71.3)	80 (75.5)	86 (74.8)	62 (70.5)
Age, years	52.2 (11.0)	49.7 (10.5)	51.2 (11.0)	50.9 (10.5)	52.1 (10.5)	50.2 (11.0)	51.6 (11.5)	50.8 (10.0)
Age categories (years), *n* (%)								
<50	41 (39.0)	44 (50.0)	47 (42.7)	38 (45.8)	34 (39.1)	51 (48.1)	47 (40.9)	42 (47.7)
≥50	64 (61.0)	44 (50.0)	63 (57.3)	45 (54.2)	53 (60.9)	55 (51.9)	68 (59.1)	46 (52.3)
Race, *n* (%)								
American Indian or Alaska native	11 (10.6)	7 (8.0)	15 (13.8)	3 (3.6)	11 (12.8)	7 (6.6)	14 (12.2)	4 (4.6)
Asian	17 (16.3)	14 (15.9)	20 (18.3)	11 (13.3)	13 (15.1)	18 (17.0)	19 (16.5)	12 (13.8)
Black or African American	5 (4.8)	2 (2.3)	5 (4.6)	2 (2.4)	4 (4.7)	3 (2.8)	7 (6.1)	3 (3.4)
White	71 (68.3)	65 (73.9)	69 (63.3)	67 (80.7)	58 (67.4)	78 (73.6)	75 (65.2)	68 (78.2)
Multiple								
Missing	1		1		1			1
BMI, kg/m^2^	37.1 (5.1)	39.6 (6.0)	37.5 (5.5)	39.2 (5.7)	38.1 (5.9)	38.3 (5.5)	37.2 (4.6)	40.0 (6.8)
BMI categories (kg/m^2^), *n* (%)								
<35	39 (37.1)	18 (20.5)	38 (34.5)	19 (22.9)	28 (32.2)	29 (27.4)	39 (33.9)	19 (21.6)
≥35 and < 40	43 (41.0)	33 (37.5)	45 (40.9)	31 (37.3)	33 (37.9)	43 (40.6)	46 (40.0)	33 (37.5)
≥40	23 (21.9)	37 (42.0)	27 (24.5)	33 (39.8)	26 (29.9)	34 (32.1)	30 (26.1)	36 (40.9)
Weight (kg)	109.9 (20.1)	119.4 (21.2)	111.8 (20.8)	117.5 (21.2)	112.3 (21.3)	115.9 (21.0)	111.3 (19.4)	119.8 (22.9)
OSA severity, *n* (%)								
Mild	1 (1.0)		1 (0.9)		1 (1.1)		1 (0.9)	
Moderate	28 (26.7)	31 (35.2)	28 (25.5)	31 (37.3)	30 (34.5)	29 (27.4)	38 (33.0)	27 (30.7)
Severe	76 (72.4)	57 (64.8)	81 (73.6)	52 (62.7)	56 (64.4)	77 (72.6)	76 (66.1)	61 (69.3)
AHI (events/hour)	49.7 (26.9)	49.8 (25.7)	49.9 (27.2)	49.7 (25.2)	46.9 (27.7)	52.1 (24.9)	45.8 (26.0)	53.7 (26.8)
ESS total score	9.0 (4.1)	11.6 (4.6)	8.7 (4.1)	12.1 (4.4)	9.0 (4.4)	11.1 (4.4)	9.8 (4.4)	10.6 (4.7)
FOSQ-10 Total score	17.3 (2.4)	14.5 (3.0)						
FOSQ Vigilance score			3.4 (0.5)	2.9 (0.7)				
FOSQ Activity Level	3.4 (0.4)	2.7 (0.6)						
SF-36v2 General Health	49.6 (8.8)	44.1 (8.8)						
SF-36v2 Vitality	52.7 (7.8)	41.6 (7.7)						
PROMIS-SRI^a^	49.8 (7.8)	61.8 (6.4)	49.9 (7.4)	62.5 (6.2)	50.6 (9.0)	59.1 (7.7)	51.5 (8.5)	60.4 (7.8)
PROMIS-SD^a^	51.7 (6.0)	61.2 (6.9)	52.1 (6.5)	61.2 (6.6)	51.5 (6.5)	59.8 (7.0)	51.0 (5.0)	62.7 (5.7)

Data are presented as mean (SD), unless specified otherwise. Grey cells indicate missing values or values not reported.

^a^T-Score using response pattern scoring.

Abbreviations: AHI, apnea-hypopnea index; BMI, body mass index; ESS, Epworth Sleepiness Scale; FOSQ, Functional Outcomes of Sleep Questionnaire; MTD, maximum tolerated dose; *n*, number of participants; *N*, total number of participants in the subgroup; PAP, positive air pressure; PGIS, Patient Global Impression of Status; PROMIS-SRI, Patient-Reported Outcomes Measurement Information System Short-Form Sleep-related Impairment 8a; PROMIS-SD, Patient-Reported Outcomes Measurement Information System Short-Form Sleep Disturbance.

### Effect of baseline fatigue on PROMs and objective assessments

#### FOSQ scores

At Week 52 in Study 1, the treatment difference between tirzepatide and placebo for improvements in FOSQ Activity Level score was greater in the Fatigued subgroup (LSM difference from placebo: 0.33) versus the Non-fatigued subgroup (LSM difference from placebo: 0.05; (Figure 1A and Supplementary Figure S1A). In Study 2, the treatment difference between tirzepatide and placebo for improvements in FOSQ Activity Level score was similar in the Fatigued (LSM difference from placebo: 0.15) and Non-fatigued subgroup (LSM difference from placebo: 0.13) at Week 52 (Figure 1A and Supplementary Figure S1A). In both studies, improvements in the FOSQ Activity Level domain score and FOSQ-10 score from baseline to Week 52 were numerically greater in the Fatigued subgroup than those in the Non-fatigued subgroup across treatment arms (Figure 1A and B).

**Figure 1:**
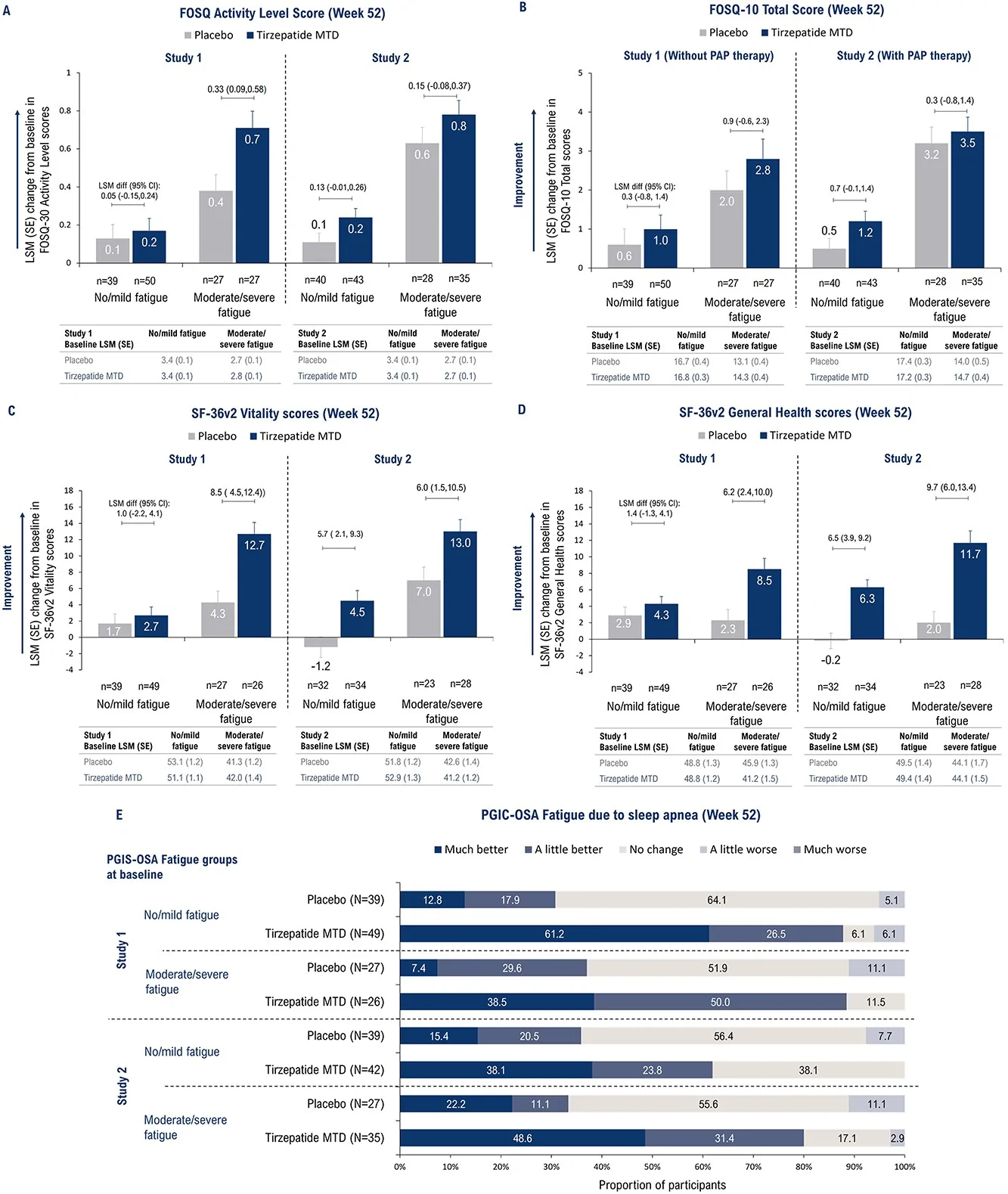
Change from baseline to Week 52 in sleep-related measures based on baseline PGIS-OSA fatigue scores. Panels A–D: Data presented are LSM change from baseline to Week 52 derived using MMRM (efficacy analysis set). Only participants with non-missing baseline value and at least one non-missing post-baseline value of the response variable were included in analysis. SF-36v2 scores are presented as norm-based scores. Panel E presents results for PGIC-OSA Fatigue assessments at Week 52 based on symptom severity at baseline. Only participants with non-missing data are included. Abbreviations: CI, confidence interval; diff, difference; FOSQ, Functional Outcomes of Sleep Questionnaire; LSM, least squares mean; MTD, maximum tolerated dose; n, number of participants; N, total number of participants in the subgroup; PAP, positive air pressure; PGIC-OSA, Patient Global Impression of Change-Obstructive Sleep Apnea; PGIS-OSA, Patient Global Impression of Symptoms-Obstructive Sleep Apnea; SE, standard error; SF-36v2, Short-Form 36 Version 2 Health Survey, Acute Form.

#### SF-36v2 scores

In Study 1, treatment difference between tirzepatide and placebo for improvements in SF-36v2 Vitality and General Health scores were greater in the Fatigued subgroup (LSM difference from placebo: Vitality: 8.5; General Health: 6.2) versus Non-fatigued subgroups (LSM difference from placebo: Vitality: 1.0; General Health: 1.4) at Week 52 (Figure 1C and D and Supplementary Figure S1C and D). In Study 2, tirzepatide treatment was associated with greater improvements in SF-36v2 Vitality and General Health scores in Fatigued and Non-fatigued subgroups versus placebo. The treatment difference between tirzepatide and placebo for improvements in these scores was similar in the Fatigued subgroup (LSM difference from placebo: Vitality: 6.0; General Health: 9.7) and Non-fatigued subgroup (LSM difference from placebo: Vitality: 5.7; General Health: 6.5) at Week 52 (Figure 1C and D and Supplementary Figure S1C and D). In both studies, improvements in the SF-36v2 Vitality and General Health domain scores from baseline to Week 52 were numerically greater in the Fatigued subgroup than those in the Non-fatigued subgroup across treatment arms (Figure 1C and D).

#### PGIC-OSA fatigue responses

A majority of participants treated with tirzepatide in the Non-fatigued subgroup reported their fatigue was “a little better/much better” at Week 52 in Study 1 (tirzepatide MTD vs. placebo: 87.8% vs. 30.8%) and Study 2 (61.9% vs. 35.9%; Figure 1E). Similarly, the majority of participants treated with tirzepatide in the Fatigued subgroup reported their fatigue was “a little better/much better” in Study 1 (tirzepatide MTD vs. placebo: 88.5% vs. 37.0%) and Study 2 (80.0% vs. 33.3%).

#### Objective assessments

In both studies, tirzepatide treatment was associated with greater improvements in AHI, body weight, SASHB, and OSA remission rates compared with placebo. The improvements across Fatigue severity subgroups were generally similar with tirzepatide treatment (Supplementary Figure S2).

### Effect of baseline sleepiness on PROMs and objective assessments

#### ESS scores

Treatment difference between tirzepatide and placebo for improvements in ESS scores was similar between the sleepiness subgroups in Study 1 (LSM difference from placebo: Non-sleepy: –1.33; Sleepy: –2.18) and Study 2 (Non-sleepy: –1.02; Sleepy: –0.57) (Figure 2A and Supplementary Figure S3A). In both studies, improvements in ESS scores from baseline to Week 52 were numerically greater in participants in the Sleepy subgroups than in the Non-sleepy subgroups across treatment arms (Figure 2A).

**Figure 2:**
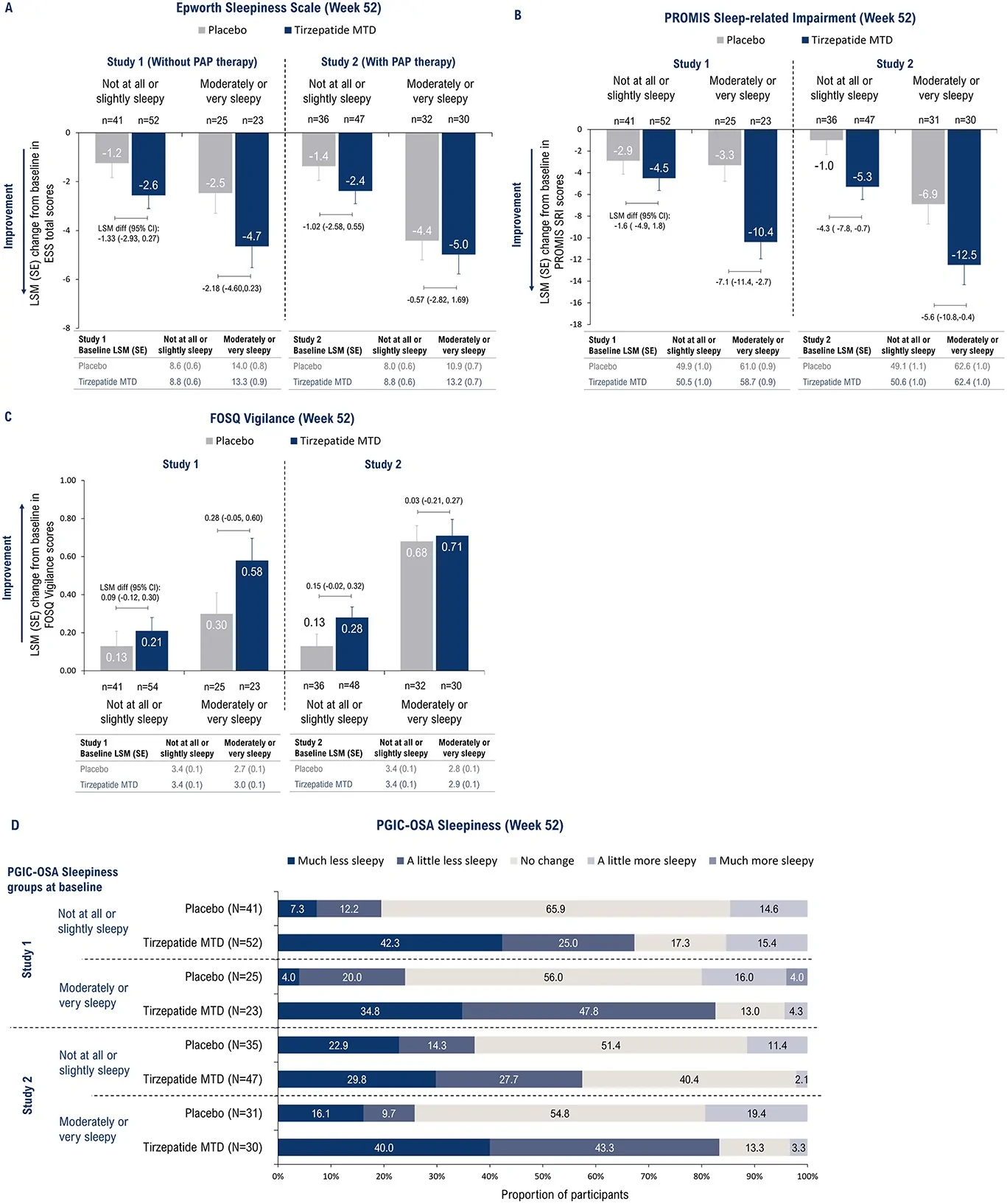
Change from baseline to Week 52 in sleep-related measures based on baseline PGIS-OSA sleepiness status. Panels A–C: Data presented are LSM change from baseline at Week 52 derived using MMRM (efficacy analysis set). Only participants with non-missing baseline value and at least one non-missing post-baseline value of the response variable were included in analysis. PROMIS-SRI scores are presented as T-scores using response pattern scoring. Panel D presents results for PGIC-OSA Sleepiness assessments at Week 52 based on symptom severity at baseline. Only participants with non-missing data are included. Abbreviations: CI, confidence interval; diff, difference; ESS, Epworth Sleepiness Scale; FOSQ, Functional Outcomes of Sleep Questionnaire; LSM, least squares mean; MTD, maximum tolerated dose; n, number of participants; N, total number of participants in the subgroup; PAP, positive air pressure; PGIC-OSA, Patient Global Impression of Change-Obstructive Sleep Apnea; PGIS-OSA, Patient Global Impression of Symptoms-Obstructive Sleep Apnea; PROMIS-SRI, Patient-Reported Outcomes Measurement Information System Short-Form Sleep-related Impairment 8a; SE, standard error.

##### PROMIS-SRI scores

In Study 1, the treatment difference between tirzepatide and placebo for improvements in PROMIS-SRI scores was greater in the Sleepy subgroup (LSM difference from placebo: –7.1) compared with the Non-sleepy subgroup (LSM difference from placebo: –1.6) at Week 52 (Figure 2B and Supplementary Figure S3B). In Study 2, tirzepatide treatment was associated with improvements in PROMIS-SRI scores in Sleepy and Non-sleepy subgroups versus placebo. The treatment difference between tirzepatide and placebo for improvements in PROMIS-SRI scores was similar in the sleepiness subgroups (LSM difference from placebo: Non-sleepy: –4.3; Sleepy: –5.6) (Figure 2B and Supplementary Figure S3B). In both studies, improvements in PROMIS-SRI scores from baseline to Week 52 were numerically greater in participants in the Sleepy subgroups than in the Non-sleepy subgroup across treatment arms (Figure 2B).

##### FOSQ vigilance scores

Treatment differences between tirzepatide and placebo groups for changes in FOSQ Vigilance scores were similar between the sleepiness subgroups in Study 1 (LSM difference from placebo: Non-sleepy: 0.09; Sleepy: 0.28) and Study 2 (Non-sleepy: 0.15; Sleepy: 0.03) (Figure 2C and Supplementary Figure S3C). In both studies, improvements in FOSQ Vigilance scores from baseline to Week 52 were numerically greater among participants in the Sleepy subgroups than in the Non-sleepy subgroup across treatment arms (Figure 2C).

##### PGIC-OSA sleepiness responses

At Week 52, majority of the participants treated with tirzepatide in the Non-sleepy subgroup reported that they were “much less/a little less” sleepy in Study 1 (tirzepatide vs. placebo: 67.3% vs. 19.5%) and Study 2 (57.5% vs. 37.2%) relative to baseline (Figure 2D). The majority of the participants treated with tirzepatide in the Sleepy subgroup reported that they were “much less/a little less” sleepy in Study 1 (tirzepatide vs. placebo: 82.6% vs. 24.0%) and Study 2 (83.3% vs. 25.8%) at Week 52 relative to baseline (Figure 2D).

##### Objective assessments

In both studies, tirzepatide treatment was associated with greater improvements in AHI, body weight, SASHB, and OSA remission rates versus placebo. The improvements in objective assessments were generally consistent across sleepiness severity subgroups with tirzepatide treatment (Supplementary Figure S4).

### Effect of baseline snoring on sleep and objective assessments

#### PGIC-OSA snoring responses

At Week 52, majority of the participants treated with tirzepatide in the Non-snoring subgroup reported that their sleep was “a little less affected/much less affected” in Study 1 (tirzepatide vs. placebo: 81.0% vs. 36.8%) and Study 2 (64.9% vs. 38.5%) relative to baseline (Figure 3). Similarly, the majority of the participants treated with tirzepatide in the Snoring subgroups reported that their sleep was “a little less affected/much less affected” in Study 1 (tirzepatide vs. placebo: 81.8% vs. 35.7%) and Study 2 (77.5% vs. 32.5%) at Week 52 relative to baseline (Figure 3).

**Figure 3:**
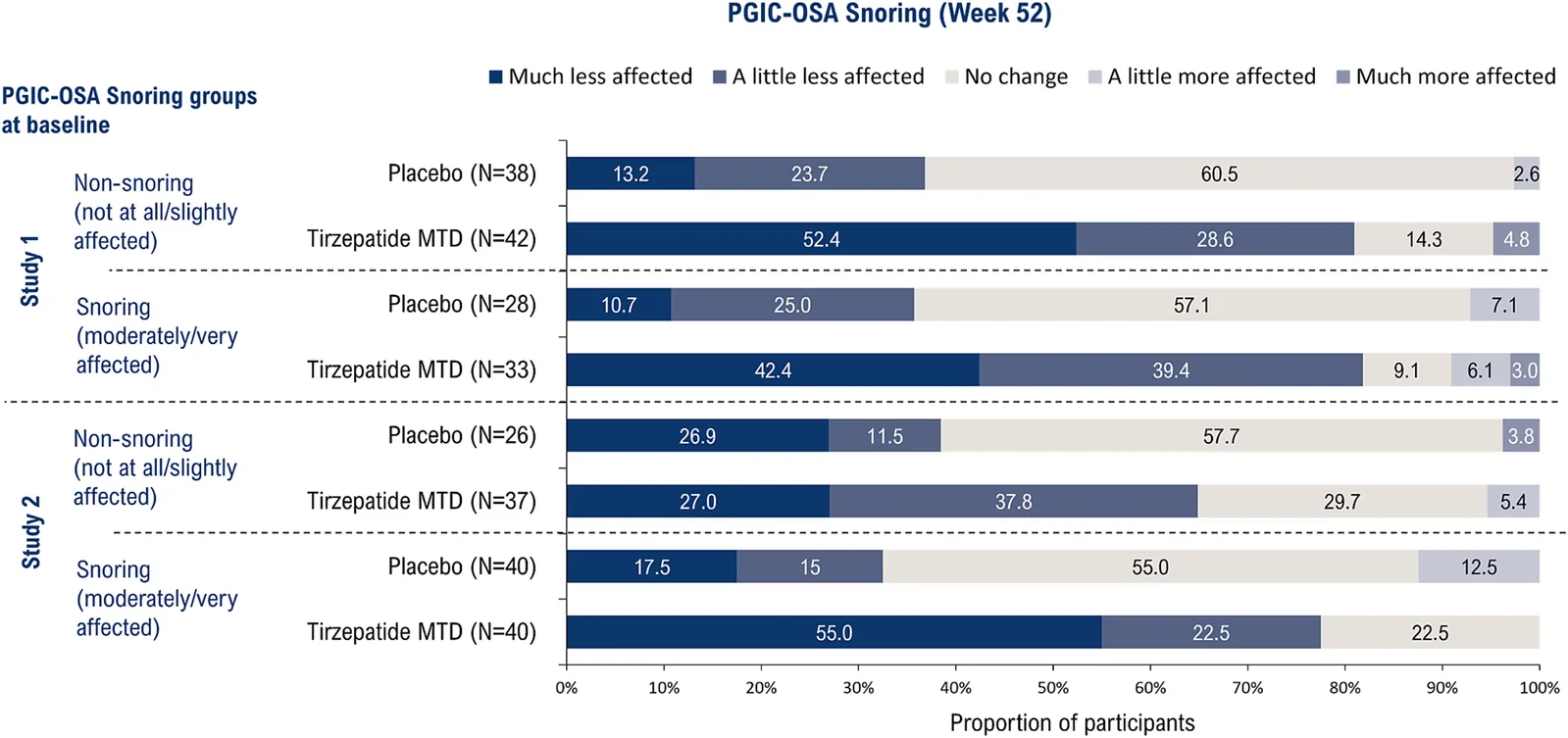
Change in PGIC-OSA Snoring scores at Week 52. PGIC-OSA Snoring assessments at Week 52 based on symptom severity at baseline. Only participants with non-missing PGIC data at week 52 were included for PGIC analyses. Abbreviations: MTD, maximum tolerated dose; N, total number of participants in the subgroup; PGIC-OSA, Patient Global Impression of Change-Obstructive Sleep Apnea.

#### Objective assessments

In both studies, tirzepatide treatment was associated with greater improvements in AHI, body weight, SASHB, and OSA remission rates compared with placebo. The improvements in objective assessments were generally similar across Snoring severity subgroups with tirzepatide treatment (Supplementary Figure S5).

### Effect of baseline sleep quality on PROMs and objective assessments

#### PROMIS-SD scores

In Study 1, treatment difference between tirzepatide and placebo groups for improvements in PROMIS-SD scores tended to be more in the Poor sleep quality (LSM difference from placebo: –5.8) versus Good sleep quality (LSM difference from placebo: –1.2) subgroups at Week 52 (Figure 4A and Supplementary Figure S6). In Study 2, the treatment difference between tirzepatide and placebo groups for improvements in PROMIS-SD scores was similar between the sleep quality subgroups at Week 52 (LSM difference from placebo: Poor sleep quality: –3.9; Good sleep quality: –4.7) (Figure 4A and Supplementary Figure S6). In both studies, improvements in PROMIS-SD scores from baseline to Week 52 were numerically greater among participants in the Poor sleep quality subgroup than in the Good sleep quality subgroup across treatment arms (Figure 4A).

**Figure 4:**
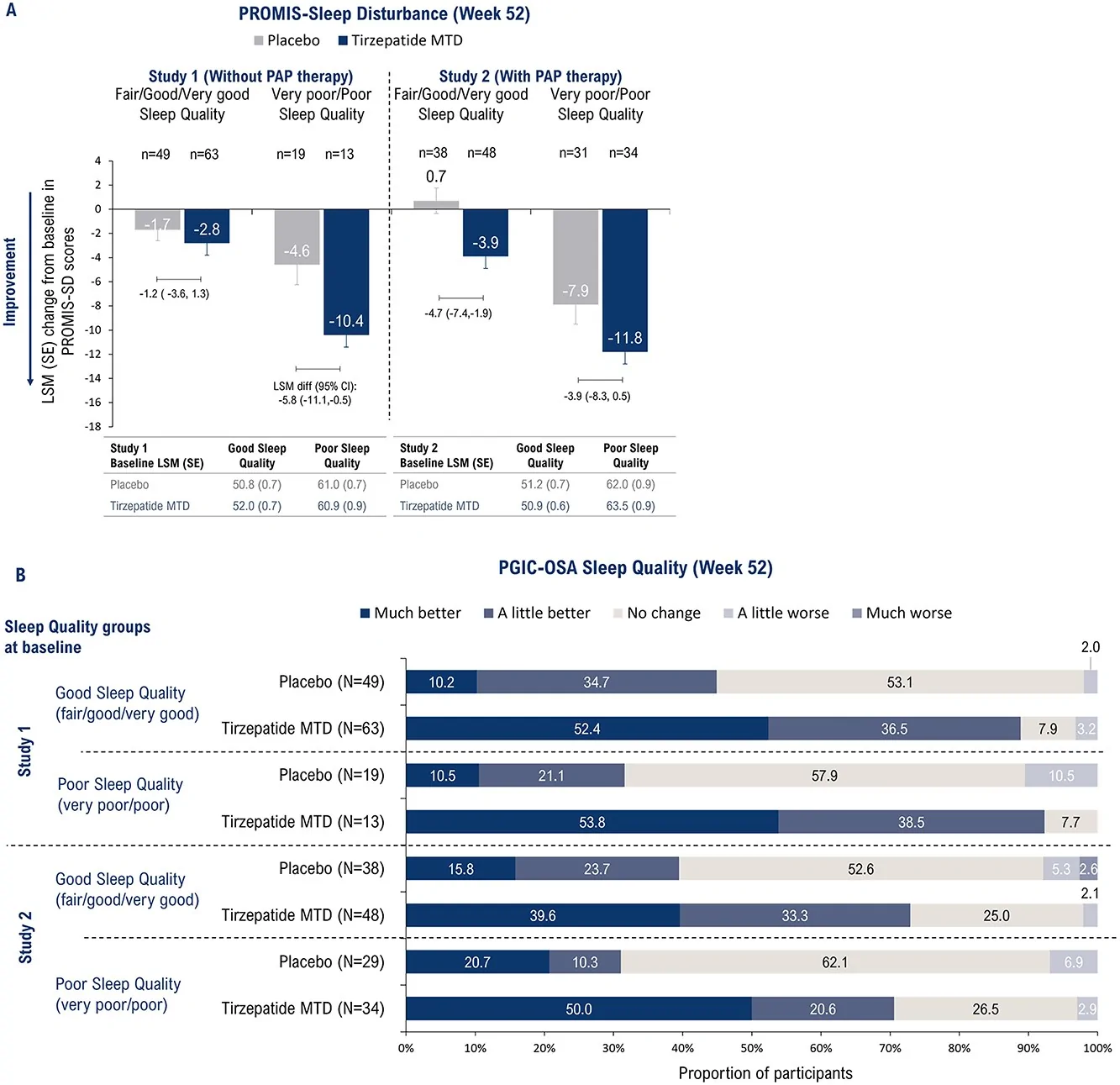
Change from baseline to Week 52 in sleep-related measures based on baseline PROMIS-Sleep Quality scores. Panel A: Data presented are LSM change from baseline to Week 52 derived using MMRM (efficacy analysis set). Only participants with non-missing baseline value and at least one non-missing post-baseline value of the response variable were included in analysis. PROMIS-SD scores are presented as T-scores using response pattern scoring. Panel B presents results for PGIC-OSA Sleep Quality assessments at Week 52 based on symptom severity at baseline. Only participants with non-missing data are included. Abbreviations: CI, confidence interval; diff, difference; LSM, least squares mean; MTD, maximum tolerated dose; n, number of participants; N, total number of participants in the subgroup; PAP, positive air pressure; PGIC-OSA, Patient Global Impression of Change-Obstructive Sleep Apnea; PROMIS-SD, Patient-Reported Outcomes Measurement Information System Short-Form Sleep Disturbance; SE, standard error.

#### PGIC-OSA sleep quality responses

At Week 52, majority of participants treated with tirzepatide in the Good sleep quality subgroup reported that their sleep quality was “a little better/much better” in Study 1 (tirzepatide vs. placebo: 88.9% vs. 44.9%) and Study 2 (72.9% vs. 39.5%) relative to baseline (Figure 4B). Similarly, majority of participants treated with tirzepatide in the Poor sleep quality subgroup reported that their sleep quality was “a little better/much better” at Week 52 in Study 1 (tirzepatide vs. placebo: 92.3% vs. 31.6%) and Study 2 (70.6% vs. 31.0%) relative to baseline (Figure 4B).

#### Objective assessments

In both studies, tirzepatide treatment was associated with greater improvements in AHI, body weight, SASHB, and OSA remission rates versus placebo. The improvements across Sleep quality subgroups were generally similar with tirzepatide treatment (Supplementary Figure S7).

Supplementary Figure S8 shows participant disposition from baseline to Week 52 by PROMs subgroup, including randomized, treated, completed, and discontinued participants.

## Discussion

In these post-hoc analyses of SURMOUNT-OSA, improvements in PROMs were consistently greater in tirzepatide groups compared to placebo. Participants with more severe symptoms at baseline reported numerically greater improvements in daytime sleep-related impairment, activity, vitality, and general health than those with less severe symptoms at baseline. Participants receiving tirzepatide also had greater improvements in AHI, body weight, SASHB, and OSA remission rates compared to those on placebo; these improvements with tirzepatide were generally similar across the baseline symptom severity subgroups. Our findings are consistent with previous analyses of PROMs among SURMOUNT-OSA participants that reported improvements in sleep quality, daily functioning, HRQoL, and OSA symptom severity following tirzepatide treatment [13, 14]. However, the previous analyses did not consider the participants’ symptom severity at baseline. The findings of the current analysis concur with previous reports indicating greater treatment benefits among people with either more severe disease or symptoms at baseline, relative to those without. For example, the 10-year Sleep AHEAD study demonstrated that greater AHI improvements over time correlated with baseline AHI [19]. Post-hoc analyses from SURMOUNT trials in people with obesity also showed that treatment with tirzepatide was associated with greater improvements in PROMs in participants who had limitations in physical functioning at baseline versus those without such limitations [20–22].

Participants in the placebo group with moderate or severe fatigue, higher levels of sleepiness, and poor sleep quality at baseline reported some improvements in related PROMs at Week 52, but these were consistently lower than those observed with tirzepatide, regardless of PAP use. Improvements in the placebo group likely resulted from lifestyle interventions (nutritional counseling, calorie restriction, and physical activity) provided to all study participants. These interventions might have positively impacted the participants’ physical condition, potentially improving their sleep-related and activity scores. Improvements in PROMs have been observed in previous studies assessing the effects of lifestyle interventions and bariatric surgery on weight [23–25] as well as other interventional studies in OSA [26, 27]. Participants treated with tirzepatide had greater improvements in objective assessments (AHI, weight, SASHB, and OSA remission) compared to those receiving placebo. Of note, even participants who reported lower symptom severity at baseline showed more improvements in objective assessments versus placebo. These results suggest that patients with moderate-to-severe OSA and obesity may derive benefit from tirzepatide treatment regardless of perceived symptoms. Furthermore, improvements in objective assessments may not always translate to corresponding improvements in PROMs. Therefore, both objective and subjective outcomes should be considered when making treatment decisions for patients with OSA and evaluating treatment effectiveness. Given the variability in symptom severity among people with OSA [28, 29], future studies can further examine baseline symptom characterization and treatment-related impact.

### Strengths and limitations

SURMOUNT-OSA was a global study with balanced demographic representation among participants. Including participants both with and without current PAP therapy in two independent studies enabled the evaluation of tirzepatide treatment effects on OSA symptoms based on baseline severity in these populations that are most frequently seen in clinical practice. Characterization of participants based on the severity of their symptoms allowed for the in-depth evaluation of tirzepatide’s effects on symptom alleviation, offering additional insights into the factors influencing patient-reported outcomes following treatment with tirzepatide. Additionally, objective assessments complemented these findings, providing a comprehensive understanding of treatment effects.

The current analysis has the following limitations. During sleep, people have limited self-awareness, which makes it challenging to obtain an accurate, objective, and complete description of sleep quality. Additionally, it is difficult to specifically ascribe functional impairment to the quality or duration of sleep versus other potentially contributing causes. Moreover, considering the day-to-day variability in sleep–wake function, evaluating sleep-related outcomes at single points in time may provide an incomplete picture compared with daily assessments. While a 7-day PAP therapy withdrawal period is generally considered sufficient, the possible carry-over effect of PAP treatment may have impacted the observed results of participants in Study 2. The comparisons between subgroups were descriptive and were not supported by statistical tests, as the small sample size may not have been adequate to detect statistically significant differences. In addition, the observed trends between or within subgroups were not formally tested with multiplicity adjustment. Therefore, the subgroup findings should be interpreted as descriptive and hypothesis-generating rather than definitive and should not be used to draw clinical conclusions regarding differential treatment effects across symptom severity subgroups. Finally, this study did not include evaluation of the clinical meaningfulness of changes in PROM scores as these have not been established in clinical practice. The Minimum Important Difference and Clinically Important Response thresholds for the PROMIS-SD, PROMIS-SRI, ESS, and/or FOSQ in the literature were primarily applied to define responders rather than to interpret mean differences between groups [14, 26, 30]. Although some outcomes showed improvements in terms of treatment difference between the tirzepatide and placebo groups, their clinical significance may require further evaluation.

## Conclusions

In the current post-hoc analyses from the SURMOUNT-OSA trials in people with moderate-to-severe obstructive sleep apnea and obesity, tirzepatide treatment was associated with greater improvements in PROMs and objective assessments compared to placebo across baseline symptom severity subgroups. Participants with moderate or severe sleepiness, fatigue, and sleep quality at baseline demonstrated greater improvements in PROMs assessing sleep disturbance, daytime sleepiness, sleep-related impairment, fatigue, and functioning compared to those with less severe symptoms. Changes in PROMs were similar across baseline snoring subgroups. In addition, improvements in objective assessments were generally similar across baseline symptom severity subgroups. These study findings suggest that patients with moderate-to-severe OSA and obesity may benefit from tirzepatide treatment regardless of perceived symptom severity.

## Supplementary Material

SLEEP-2025-0943_Supplementary_Material_zsag184

## Data Availability

Eli Lilly and Company provides access to all individual participant data collected during the trial, after anonymization, except for pharmacokinetic or genetic data. Data are available to request 6 months after the indication studied has been approved in the USA and European Union and after primary publication acceptance, whichever is later. No expiration date for data requests is currently set once data have been made available. Access is provided after a proposal has been approved by an independent review committee identified for this purpose and after receipt of a signed data-sharing agreement. Data and documents, including the study protocol, statistical analysis plan, clinical study report, and blank or annotated case report forms, will be provided in a secure data-sharing environment. For details on submitting a request, see the instructions provided at www.vivli.org.
