# Supplementary material for: Changes in patient-reported outcomes and objective assessments based on baseline symptom severity in SURMOUNT-OSA: post-hoc analyses
Source: Sleep. 2026 Jul 7;49(10):zsag184. doi: 10.1093/sleep/zsag184 (PMC13658257; doi:10.1093/sleep/zsag184)
Supplement: Supplementary file 1 [file sleep-2025-0943_supplementary_material_zsag184.docx]

# **Title**

**Changes in patient-reported outcomes and objective assessments based on baseline symptom severity in SURMOUNT-OSA: Post hoc analyses.**

**Authors:**

Chisom Kanu^1,^*, Shraddha Shinde^1^, Beverly Falcon^1^, Josef Bednarik^1^, Cathy Chang Xie^1^, Kevin Trice^1^, Terri Weaver^2^

**Affiliations**

^1^Eli Lilly and Company, Indianapolis, IN, USA

^2^College of Nursing, University of Illinois Chicago, Chicago, IL, USA; University of Pennsylvania School of Nursing, Philadelphia, PA, USA

***Corresponding author**

Chisom Kanu

Eli Lilly and Company,

893 S. Delaware Street

Indianapolis, IN, 46285, USA

e-mail: [kanu_chisom@lilly.com](mailto:kanu_chisom@lilly.com)

ORCID iD: 0000-0002-2621-8652

**Supplementary material**

Supplementary Table 1: Description of PROMs

Supplementary Figure 1: Treatment differences in sleep-related measures based on baseline PGIS-OSA fatigue scores

Supplementary Figure 2: Change from baseline at Week 52 in objective assessments based on baseline PGIS-OSA Fatigue scores

Supplementary Figure 3: Treatment differences in sleep-related measures based on baseline PGIS-OSA sleepiness status

Supplementary Figure 4: Change from baseline at Week 52 in objective assessments based on baseline PGIS-OSA Sleepiness status

Supplementary Figure 5: Change from baseline at Week 52 in objective assessments based on baseline PGIC-OSA Snoring status

Supplementary Figure 6: Treatment differences in sleep-related measures based on baseline PROMIS-Sleep Quality scores

Supplementary Figure 7: Change from baseline at Week 52 in objective assessments based on baseline PROMIS-Sleep Quality score

Supplementary Figure 8: Patient disposition from baseline to week 52 by subgroups

Supplementary methods: MMRM and Logistic regression

**Supplementary Table 1: Description of PROMs**

| **Symptom scale/patient-reported outcomes** | **Description** |
| --- | --- |
| PGIS-OSA Fatigue | Single-item, participant self-rated assessment of their overall level of fatigue due to OSA, “over the past 7 days.” The item is rated on a 4-point scale ranging from “No fatigue” to “Severe fatigue.” |
| PGIS-OSA Sleepiness | This is a single-item, participant self-rated assessment of their overall level of sleepiness due to OSA during waking hours, “over the past 7 days.” The item is rated on a 4-point scale ranging from “Not at all sleepy” to “Very sleepy.” |
| PGIS-OSA Snoring | The PGIS-OSA Snoring scale consists of two items. The first item is a participant self-rated assessment of their overall perception of the severity of their snoring due to OSA, “over the past 7 days,” with respect to how much their snoring has affected their sleep. The item is rated on a 4-point scale ranging from “Not at all affected” to “Very affected.” For the second item, participants will be asked on a 3-point scale (“Not at all” to “All the time”) if they have ever been told by someone else that they snore in their sleep. |
| Epworth Sleepiness Scale (ESS) | The ESS is an 8-item participant-completed measure that asks the participant to rate on a scale of 0 (would never doze) to 3 (high chance of dozing), their usual chances of dozing in 8 different daytime situations, with a recall period of “in recent times.” The ESS total score is the sum of the 8-item scores and ranges from 0 to 24, with higher scores indicating greater daytime sleepiness. |
| PROMIS-Sleep-related Impairment | The PROMIS Short-Form v1.0 Sleep-related Impairment (SRI) 8a assesses self-reported perceptions of alertness, sleepiness, and tiredness during usual waking hours, and the perceived functional impairments associated with sleep problems or impaired alertness. The PROMIS Short-Form v1.0 Sleep-related Impairment 8a consists of 8 items each rated on a 5-point scale ranging from “not at all” to “very much.” Items have a recall period of “in the past 7 days.” Individual item scores are added to obtain a raw score, with higher scores indicating more sleep-related Impairment. Raw scores can be converted to a T-score, which is standardized with a mean of 50 and a SD of 10. |
| PROMIS-Sleep Disturbance | The PROMIS Short-Form v1.0 Sleep Disturbance 8b assesses self-reported perceptions of sleep quality, sleep depth, and restoration associated with sleep, including perceived difficulties and concerns with getting to sleep or staying asleep, as well as perceptions of the adequacy of and satisfaction with sleep. The PROMIS Short-Form v1.0 Sleep Disturbance 8b consists of 8 items each rated on a 5-point scale ranging from “not at all” to “very much,” “never” to “always,” or “very poor” to “very good.” Items have a recall period of “in the past 7 days.” Individual item scores are added to obtain a raw score, with higher scores indicating more sleep disturbance. Raw scores can be converted to a T-score, which is standardized with a mean of 50 and a SD of 10. |
| Functional Outcomes of Sleep Questionnaire (FOSQ) | The FOSQ is a 30-item sleep-specific, participant-completed questionnaire used to assess the effect of disorders associated with excessive daytime sleepiness (EDS) on daily functioning in adults. It assesses the following 5 domains of  • General productivity (8 items)  • Activity level (9 items)  • Vigilance (7 items)  • Social outcomes (2 items)  • Intimate and sexual relationships (4 items)  The FOSQ items assess participant’s current status with each item rated on a scale of 1 (extreme difficulty) to 4 (no difficulty), with an additional not applicable (0 = “I don’t do this activity for other reasons”) also available. Individual domain scores are calculated by taking the mean of answered, non-zero items within each domain and a total score can be calculated by first computing the mean score for each domain, then multiplying the mean of the domain scores by 5. The Total score for the FOSQ 10-item short-form (FOSQ-10) can also be calculated. |
| PGIC-OSA Sleepiness | This is a single-item, participant self-rated assessment of the change in their overall level of sleepiness due to OSA during waking hours, “since you started taking the study medication.” The item is rated on a 5-point scale ranging from “Much more sleepy” to “Much less sleepy.” |
| PGIC-OSA Snoring | This is a single-item, participant self-rated assessment of the overall change in how their snoring has affected their sleep, “since you started taking the study medication.” The item is rated on a 5-point scale ranging from “My sleep is much more affected” to “My sleep is much less affected.” |
| PGIC-OSA Sleep Quality | This is a single-item, participant self-rated assessment of the change in their overall sleep quality due to OSA, “since you started taking the study medication.” The item is rated on a 5-point scale ranging from “Much worse” to “Much better.” |
| PGIC-OSA Fatigue | This is a single-item, participant self-rated assessment of the change in their overall level of fatigue due to OSA, “since you started taking the study medication.” The item is rated on a 5-point scale ranging from “Much worse” to “Much better.” |
| Short-Form 36 Version 2 Health Survey, Acute Form, 1-week Recall Version | The SF-36v2 will be included to assess health-related quality of life from baseline to Week 52. The SF-36v2 acute form, 1-week recall version is a 36-item generic, participant-completed measure designed to assess the following 8 domains over “the past week.”  Domains: Physical Functioning, Role-Physical, Bodily Pain, General Health, Vitality, Social Functioning, Role-Emotional, and Mental Health  The Physical Functioning domain assesses limitations due to health “now” while the remaining domains assess functioning “in the past week.” Each domain is scored individually and information from these 8 domains are further aggregated into 2 health component summary scores: Physical Component Summary and Mental Component Summary. Items are answered on Likert scales of varying lengths (3-point, 5-point, or 6-point scales). Scoring of each domain and both summary scores are norm-based and presented in the form of T-scores, with a mean of 50 and SD of 10; higher scores indicate better levels of function and/or better health. |

Abbreviations: PGIC, Patient Global Impression of Change; OSA, Obstructive Sleep Apnea; PROMIS-SRI, Patient-Reported Outcomes Measurement Information System Short-Form Sleep-related Impairment 8a; PROMIS-SD, Patient-Reported Outcomes Measurement Information System Short-Form Sleep Disturbance; SF-36v2, Short-Form 36 Version 2 Health Survey, Acute Form. Internal consistency reliability estimates (e.g., Cronbach’s alpha) were not calculated for the current analyses because item-level questionnaire responses were not available; single-item measures do not have internal consistency estimates.

**Supplementary Figure 1: Treatment differences in sleep-related measures based on baseline PGIS-OSA fatigue scores**

Data presented are LSM change difference (95% CI) derived using mixed model repeated measures (efficacy analysis set). Only participants with non-missing baseline value and at least one non-missing post-baseline value of the response variable were included in analysis. SF-36v2 scores are presented as norm-based scores.

Abbreviations: CI, confidence interval; diff, difference; FOSQ, Functional Outcomes of Sleep Questionnaire; LSM, least squares mean; PAP, positive air pressure, PGIC-OSA, Patient Global Impression of Change-Obstructive Sleep Apnea; PGIS-OSA, Patient Global Impression of Symptoms-Obstructive Sleep Apnea; SF-36v2, Short-Form 36 Version 2 Health Survey Acute Form.

**Supplementary Figure 2: Change from baseline at Week 52 in objective assessments based on baseline PGIS-OSA Fatigue scores**

|  |   **B** |
| --- | --- |
|  |  |
|  |  |
|  |  |
|  |  |
|   Panels A-D: Only participants with non-missing baseline PGIS Fatigue scores are included. Data from the efficacy analysis set was utilized for the analysis.  Panel A: LSM percent change from baseline in AHI from MMRM. Covariates include baseline value, geographic region (US/outside of US), sex, treatment, time, and the interaction between treatment and time.  Panel B: LSM percent change from baseline in body weight from MMRM. Covariates include baseline value, geographic region, baseline OSA severity (not severe: AHI <30 events/hour; severe: AHI ≥30 events/hour), sex, treatment, time, and the interaction between treatment and time.  Panel C: Model-based estimate (MBE) percent change from baseline in SASHB from MMRM with log-transformation. Covariates include log (Baseline) value, geographic region, baseline OSA severity, sex, treatment, time, and the interaction between treatment and time  Panel D: Proportion of participants achieving OSA remission estimated after imputing missing AHI data at Week 52 using the multiple imputation method. The odds ratio for remission was derived from logistic regression with the imputed datasets after controlling for baseline AHI, geographic region, sex and treatment. The results are pooled using Rubin’s rules.  Abbreviations: AHI, apnea-hypopnea Index; CI, confidence interval; diff, difference; LSM, least squares mean; MMRM, mixed model repeated measures; MTD, maximum tolerated dose; OSA, obstructive sleep apnea; PGIS-OSA, Patient Global Impression of Symptoms-Obstructive Sleep Apnea; SASHB, sleep apnea-specific hypoxic burden; SE, standard error. | |

**Supplementary Figure 3: Treatment differences in in sleep-related measures based on baseline PGIS-OSA sleepiness status**

Data presented are LSM change difference (95% CI) derived using mixed model repeated measures (efficacy analysis set). Only participants with non-missing baseline value and at least one non-missing post-baseline value of the response variable were included in analysis. PROMIS-SRI scores are presented as T-scores using response pattern scoring.

Abbreviations: CI, confidence interval; diff, difference; FOSQ, Functional Outcomes of Sleep Questionnaire; LSM, least squares mean; PAP, positive air pressure; PGIS-OSA, Patient Global Impression of Symptoms-Obstructive Sleep Apnea; PROMIS-SRI, Patient-Reported Outcomes Measurement Information System Short-Form Sleep-related Impairment 8a.

**Supplementary Figure 4: Change from baseline at Week 52 in objective assessments based on baseline PGIS-OSA Sleepiness status**

|  |  |
| --- | --- |
|  |  |
|  |  |
|  |  |
|       Panels A-D: Only participants with non-missing baseline PGIS Sleepiness scores are included. Data from the efficacy analysis set was utilized for the analysis.  Panel A: LSM percent change from baseline in AHI from MMRM. Covariates include baseline value, geographic region (US/outside of US), sex, treatment, time, and the interaction between treatment and time.  Panel B: LSM percent change from baseline in body weight from MMRM. Covariates include baseline value, geographic region, baseline OSA severity (not severe: AHI <30 events/hour; severe: AHI ≥30 events/hour), sex, treatment, time, and the interaction between treatment and time.  Panel C: Model-based estimate (MBE) percent change from baseline in SASHB from MMRM with log-transformation. Covariates include log (Baseline) value, geographic region, baseline OSA severity, sex, treatment, time, and the interaction between treatment and time  Panel D: Proportion of participants achieving OSA remission estimated after imputing missing AHI data at Week 52 using the multiple imputation method. The odds ratio for remission was derived from logistic regression with the imputed datasets after controlling for baseline AHI, geographic region, sex and treatment. The results are pooled using Rubin’s rules.  Abbreviations: AHI, apnea-hypopnea Index; CI, confidence interval; diff, difference; LSM, least squares mean; MMRM, mixed model repeated measures; MTD, maximum tolerated dose; OSA, obstructive sleep apnea; PGIS-OSA, Patient Global Impression of Symptoms-Obstructive Sleep Apnea; SASHB, sleep apnea-specific hypoxic burden; SE, standard error. | |

**Supplementary Figure 5: Change from baseline at Week 52 in objective assessments based on baseline PGIC-OSA Snoring status**

|  |  |
| --- | --- |
|  |  |
|  |  |
|  |  |
|   Panels A-D: Only participants with non-missing baseline PGIS Snoring scores are included. Data from the efficacy analysis set was utilized for the analysis.  Panel A: LSM percent change from baseline in AHI from MMRM. Covariates include baseline value, geographic region (US/outside of US), sex, treatment, time, and the interaction between treatment and time.  Panel B: LSM percent change from baseline in body weight from MMRM. Covariates include baseline value, geographic region, baseline OSA severity (not severe: AHI <30 events/hour; severe: AHI ≥30 events/hour), sex, treatment, time, and the interaction between treatment and time.  Panel C: Model-based estimate (MBE) percent change from baseline in SASHB from MMRM with log-transformation. Covariates include log (Baseline) value, geographic region, baseline OSA severity, sex, treatment, time, and the interaction between treatment and time  Panel D: Proportion of participants achieving OSA remission estimated after imputing missing AHI data at Week 52 using the multiple imputation method. The odds ratio for remission was derived from logistic regression with the imputed datasets after controlling for baseline AHI, geographic region, sex and treatment. The results are pooled using Rubin’s rules.  Abbreviations: AHI, apnea-hypopnea Index; CI, confidence interval; diff, difference; LSM, least squares mean; MMRM, mixed model repeated measures; MTD, maximum tolerated dose; OSA, obstructive sleep apnea; PGIS-OSA, Patient Global Impression of Symptoms-Obstructive Sleep Apnea; SASHB, sleep apnea-specific hypoxic burden; SE, standard error. | |

**Supplementary Figure 6: Treatment differences in sleep-related measures based on baseline PROMIS-Sleep Quality scores**

Data presented are LSM change difference (95% CI) derived using mixed model repeated measures (efficacy analysis set). Only participants with non-missing baseline value and at least one non-missing post-baseline value of the response variable were included in analysis. PROMIS-SD scores are presented as T-scores using response pattern scoring.

Abbreviations: CI, confidence interval; diff, difference; LSM, least squares mean; PAP, positive air pressure, PROMIS-SD, Patient-Reported Outcomes Measurement Information System Short-Form Sleep Disturbance.

**Supplementary Figure 7: Change from baseline at Week 52 in objective assessments based on baseline PROMIS-Sleep Quality scores**

|  |  |
| --- | --- |
|  |  |
|  |  |
|  |  |
| Panels A-D: Only participants with non-missing baseline PGIS Sleep Quality scores are included. Data from the efficacy analysis set was utilized for the analysis.  Panel A: LSM percent change from baseline in AHI from MMRM. Covariates include baseline value, geographic region (US/outside of US), sex, treatment, time, and the interaction between treatment and time.  Panel B: LSM percent change from baseline in body weight from MMRM. Covariates include baseline value, geographic region, baseline OSA severity (not severe: AHI <30 events/hour; severe: AHI ≥30 events/hour), sex, treatment, time, and the interaction between treatment and time.  Panel C: Model-based estimate (MBE) percent change from baseline in SASHB from MMRM with log-transformation. Covariates include log (Baseline) value, geographic region, baseline OSA severity, sex, treatment, time, and the interaction between treatment and time  Panel D: Proportion of participants achieving OSA remission estimated after imputing missing AHI data at Week 52 using the multiple imputation method. The odds ratio for remission was derived from logistic regression with the imputed datasets after controlling for baseline AHI, geographic region, sex and treatment. The results are pooled using Rubin’s rules.  Abbreviations: AHI, apnea-hypopnea Index; CI, confidence interval; diff, difference; LSM, least squares mean; MMRM, mixed model repeated measures; MTD, maximum tolerated dose; OSA, obstructive sleep apnea; PGIS-OSA, Patient Global Impression of Symptoms-Obstructive Sleep Apnea; SASHB, sleep apnea-specific hypoxic burden; SE, standard error. | |
|  | |

**Supplementary Figure 8: Patient disposition from baseline to Week 52 by subgroups**

Disposition shown includes randomized, treated, completed, discontinued study period and study treatment. N is the number of subjects randomized; n is the number of subjects with non-missing baseline PRO for grouping.

Abbreviations: OSA, obstructive sleep apnea; PAP, positive air pressure; PRO, patient-reported outcomes.

**Supplementary methods:**

All analyses described below were conducted separately within each PRO subgroup defined by baseline symptom severity (PGIS-OSA fatigue score, PGIS-OSA sleepiness status, PGIC-OSA snoring, and PROMIS-Sleep Quality scores), using the corresponding subgroup-specific analysis set.

**Mixed model repeated measures (MMRM)**

Continuous longitudinal endpoints were analyzed using an MMRM including all available scheduled post-baseline observations within the efficacy analysis set (excluding observations after study treatment discontinuation; and for Study 1, excluding observations after PAP initiation). Models were fit using restricted maximum likelihood (REML). Fixed effects included treatment group, visit, treatment-by-visit interaction, geographic region (US vs outside the US), and sex, with baseline value of the endpoint as a continuous covariate; baseline OSA severity stratum (not severe vs severe) was additionally included for endpoints other than AHI. An unstructured within-participant covariance matrix was used; if the model failed to converge, covariance structures were evaluated sequentially (Toeplitz heterogeneous, autoregressive heterogeneous, compound symmetry heterogeneous, Toeplitz, autoregressive, compound symmetry). Least squares means and standard errors were obtained from the fitted model, and treatment effects at Week 52 were summarized as least squares mean (or model-based estimate) differences versus placebo with 95% confidence intervals.

**Logistic regression**

For binary Week 52 endpoints (e.g., OSA remission/mild non-symptomatic OSA or ≥50% AHI reduction), endpoints were analyzed using logistic regression including treatment, geographic region (US vs outside the US), sex, and baseline AHI as covariates; results were summarized as odds ratios with 95% confidence intervals. For analyses involving imputation, missing Week 52 AHI values were imputed first using multiple imputation, and results were pooled using Rubin’s rules.
